# Multi-Disciplinary Management of Athletes with Post-Concussion Syndrome: An Evolving Pathophysiological Approach

**DOI:** 10.3389/fneur.2016.00136

**Published:** 2016-08-24

**Authors:** Michael J. Ellis, John Leddy, Barry Willer

**Affiliations:** ^1^Pan Am Concussion Program, Section of Neurosurgery, Department of Surgery, Pediatrics and Child Health, University of Manitoba, Children’s Hospital Research Institute of Manitoba, Canada North Concussion Network, Winnipeg, MB, Canada; ^2^UBMD Department of Orthopaedics and Sports Medicine, State University of New York at Buffalo Jacobs School of Medicine and Biomedical Sciences, Buffalo, NY, USA; ^3^Department of Psychiatry, State University of New York at Buffalo Jacobs School of Medicine and Biomedical Sciences, Buffalo, NY, USA

**Keywords:** sports-related concussion, post-concussion syndrome, athlete, multi-disciplinary, management, treatment

## Abstract

Historically, patients with sports-related concussion (SRC) have been managed in a uniform fashion consisting mostly of prescribed physical and cognitive rest with the expectation that all symptoms will spontaneously resolve with time. Although this approach will result in successful return to school and sports activities in the majority of athletes, an important proportion will develop persistent concussion symptoms characteristic of post-concussion syndrome (PCS). Recent advances in exercise science, neuroimaging, and clinical research suggest that the clinical manifestations of PCS are mediated by unique pathophysiological processes that can be identified by features of the clinical history and physical examination as well as the use of graded aerobic treadmill testing. Athletes who develop PCS represent a unique population whose care must be individualized and must incorporate a rehabilitative strategy that promotes enhanced recovery of concussion-related symptoms while preventing physical deconditioning. In this review, we present our evolving evidence-based approach to evaluation and management of athletes with PCS that aims to identify the pathophysiological mechanisms mediating persistent concussion symptoms and guides the initiation of individually tailored rehabilitation programs that target these processes. In addition, we outline the important qualified roles that multi-disciplinary healthcare professionals can play in the management of this patient population, and discuss where future research efforts must be focused to further evaluate this evolving pathophysiological approach.

## Background

Over the past 10–15 years, clinical studies have established sports-related concussion (SRC) as a form of traumatic brain injury (TBI) that results in temporary alterations in neurological and neurocognitive functioning that typically recover within 1–2 weeks post-injury (1–4). In a subset of adult and pediatric SRC patients, however, conservative management will fail to result in clinical recovery and patients will develop prolonged symptoms characteristic of post-concussion syndrome (PCS). Historically, PCS patients were managed clinically with the assumption that persistent concussion symptoms were representative of an ongoing global metabolic brain injury that required additional amounts of cognitive and physical rest (5, 6). While this approach may lead to symptom resolution in a small proportion of these patients, it is inappropriate for athletes and does not take into account the pathophysiological processes that govern the heterogeneous clinical manifestations of PCS.

To address these limitations, emerging clinical and laboratory research suggests that features of the clinical history and physical examination, along with the use of supplementary tests such as graded aerobic treadmill testing, can be used to provide a view into unique pathophysiological mechanisms responsible for the persistent symptoms of PCS and help guide the initiation of targeted rehabilitative therapies leading to enhanced clinical recovery and successful return to sports (7–11). Although previous reports have examined treatment options for patients with concussion (12–14), a comprehensive evidence-based pathophysiology-based treatment approach to direct multi-disciplinary management of PCS in athletes is lacking.

Accordingly, we present here an evolving clinical approach to athletes with PCS that focuses on early identification and targeted treatment of the pathophysiological mechanisms governing persistent concussion symptoms. This report also outlines the qualified roles and responsibilities of healthcare professionals that can help contribute to the multi-disciplinary management of this unique patient population.

## The Natural History of Sports-Related Concussion

In order to properly manage athletes with PCS, physicians must have an understanding of the natural history of SRC and the clinical factors associated with prolonged recovery and the development of PCS. Despite receiving enormous research attention over the past two decades, our understanding of what constitutes a normal clinical recovery following SRC continues to evolve. At present, expert opinion supports the understanding that SRC is a self-limiting condition that presents with symptoms and alterations in neurocognitive and neurological functioning that typically resolve within 7–10 days with proper return-to-learn and return-to-play management (3). This opinion is supported by epidemiological studies and systematic reviews performed largely in collegiate athletes that document symptom recovery as well as normalization of selected balance and neurocognitive scores within this short time frame (1, 15, 16). The proportion of adult athletes that experience persistent symptoms beyond this period varies in the literature from 10 to 30% (12). In contrast to collegiate and professional athletes, there is some evidence to suggest that child and adolescent athletes take longer to return to their neurological baseline following SRC. A recent systematic review and meta-analysis concluded that high school athletes demonstrate neurocognitive recovery within 7 days and symptomatic recovery within 15 days post-injury (4). These figures likely represent the true natural history of SRC; however, longer recovery times are commonly observed among patients who present to pediatric tertiary concussion clinics, where median lengths of recovery range from 25 to 75 days and 43–73% of patients experience symptoms lasting greater than 4 weeks (17–19).

Given our evolving understanding of what constitutes a normal recovery following SRC, predicting how long it will take for patients to recover and determining which patients are at risk of developing PCS represent significant clinical challenges. Clinical variables associated with prolonged recovery and the development of PCS vary across studies but may include younger age, female sex, loss of consciousness or post-traumatic amnesia at the time of injury, a previous history of concussion, ADHD and mood disorders, initial headache or dizziness at the time of injury, delayed symptom onset, and initial symptom burden (2, 18, 20–28). In addition to these clinical factors, emerging work also suggests that pre-injury psychological factors such as somatization (29) and resilience (30) may also impact post-injury functioning. Although an appreciation of these clinical factors may aid physicians in their ability to provide anticipatory guidance to patients and their parents (31), more recent research has focused on identifying clinical biomarkers that reflect the pathophysiological processes governing persistent symptoms. For instance, emerging evidence suggests that patients who present with subjective and objective evidence of vestibulo-ocular dysfunction take twice as long to recover compared with those without these findings and are more likely to develop PCS (19).^1^ Likewise, acute concussion patients who demonstrate significant exercise intolerance on graded aerobic treadmill testing take longer to recover than those with less exercise intolerance (Leddy et al., unpublished data).

Taken together, the vast majority of pediatric and adult SRC patients will achieve a complete neurological recovery and return to full school and sports activities within weeks of injury. However, those that experience persistent symptoms will benefit from early identification and further multi-disciplinary assessment by a team of experts with clinical training in TBI.

## Defining Post-Concussion Syndrome in Athletes

One of the obstacles to evaluating the effect of PCS on patient outcomes and deciding when to initiate treatment in SRC patients is the lack of a universal clinically accepted definition for PCS. At present, the two standardized definitions of PCS have shortcomings that limit their application to clinical practice. The Diagnostic and Statistics Manual (DSM-IV) defines PCS as: (1) cognitive deficits in attention or memory and; (2) at least three or more symptoms including; headache, dizziness, fatigue, irritability, apathy, personality change, or sleep or affective disturbance (32). To meet the DSM criteria for PCS, symptoms must be present for three months or greater. In comparison, the World Health Organization’s International Classification of Diseases (ICD-10) defines PCS as the presence of three or more of the following symptoms that must be present within the first month post-injury including: headache, dizziness, fatigue, irritability, insomnia, and concentration or memory difficulty. One of the well acknowledged limitations of the existing definitions of PCS is their poor specificity and their inclusion of symptoms that are frequently experienced by patients with other neurological disorders such as depression and migraine as well healthy children and adolescents during everyday life (33, 34). Studies suggest that there is little agreement between these definitions in clinical practice (35) and that the ICD-10 definition is six times more sensitive than the DSM definition (32). In addition to these standardized definitions, other non-standardized definitions such as prolonged symptoms or recovery (symptoms lasting greater than 7–28 days) (2, 23), persistent postconcussion symptoms (36), and delayed symptom resolution (37) have also been used to describe PCS patients. Such descriptions have not only created confusion in the literature but limit comparisons between these studies. Although standardized definitions of PCS are important for research purposes, many athletes with symptoms persisting weeks after injury will not meet these PCS criteria yet experience significant impairments in quality of life, including feelings of worry and hopelessness regarding the effect of their injury on return to school and sport activities. Importantly, many athletes also begin to experience aerobic deconditioning within 1–2 weeks of inactivity that can also manifest as fatigue, irritability, and sleep disturbance that can be difficult to distinguish from symptoms of PCS. Taken together, many athletes will not meet any standardized criteria for PCS; however, early active rehabilitation should be considered in those with symptoms persisting beyond 1.5–2 weeks to avoid aerobic deconditioning and help optimize neurological recovery.

## Pathophysiology of Acute SRC and PCS

Basic science and neuroimaging research continues to shape our understanding of the pathophysiological mechanisms that govern clinical symptoms in acute concussion and PCS. Historically, the clinical manifestations of concussion were thought to arise primarily as a result of functional disturbances in brain physiology rather than a structural brain injury (3). However, recent research has begun to challenge this assumption. Animal models of concussion and TBI suggest that the acute phase of injury is primarily characterized by an increase in cerebral cellular energy demand coupled by insufficient energy substrate delivery resulting in a metabolic energy crisis (38). This metabolic mismatch occurs during a period in which alterations in neuronal depolarization, ion transport, glycolysis, mitochondrial function, and excitatory neurotransmitter release are met with reductions in global and regional cerebral blood flow (CBF). Although the temporal course of these metabolic processes has been well documented in animal studies, the magnitude and duration of these pathophysiological alterations in humans remain poorly understood. Among the secondary brain injury processes that play an important role in the pathophysiology of moderate, and severe TBI is ischemia. Studies suggest that inadequate CBF delivery during acute TBI is an independent risk factor for poor outcome and death (39–42) while 90% of fatal TBI patients demonstrate histological evidence of ischemia at autopsy (43, 44). That patients often report an exacerbation of concussion symptoms with increased physical and cognitive exercise suggests that a mismatch between cerebral metabolic demand and CBF delivery may potentially mediate some of the symptoms of acute SRC and PCS. Recent studies using magnetic resonance imaging (MRI)-based quantitative CBF sequences have demonstrated global and regional reductions in resting CBF in adolescents and collegiate athletes with SRC (45–48). Despite these findings, the relationship between exercise intolerance and CBF dysregulation in concussion remains poorly understood but may be mediated by alterations in autonomic nervous system function. Concussion patients have been found to demonstrate physiological markers of increased sympathetic nervous system output such as higher resting heart rates and blood pressure (49). Furthermore, preliminary evidence suggests that concussion is associated with CBF dysregulation that is mediated through alterations in carbon dioxide sensitivity (50) and cerebrovascular reactivity (51–54). Given that mean arterial blood pressure and PaCO_2_ are important mediators of global and regional CBF, it is possible that dysfunction within these systems is responsible for some of the clinical manifestations of acute SRC and PCS.

In addition to functional changes in neurovascular physiology, accumulating studies suggest that acute SRC may also be associated with microstructural white matter injury. Studies using diffusion tensor imaging (DTI) have detected group differences in several quantitative DTI indices between SRC patients and healthy controls (55–57); however, the proportion of SRC patients who develop these changes remains unclear as does the relationship of these changes to the severity and volume of head injuries, underlying neuropathology, as well as clinical outcomes. Evidence also suggests that concussion and mTBI are associated with the release of proteins such as S100β, glial fibrillary acidic protein, tau, and αII-spectrin N-terminal fragment (SNTF) (58–62). Although preliminary studies suggest these proteins can be readily detected in the peripheral blood and cerebrospinal fluid of concussion and mTBI patients, future work is needed to establish the clinical utility of these potential biomarkers in individual patient management.

While these research advances support the notion that acute SRC is mediated by alterations in brain cellular and cerebrovascular physiology, the extent to which these processes mediate the clinical manifestations of PCS remains unclear. Clinically, some symptoms of PCS appear reflective of a persistent global metabolic brain injury while others point to isolated dysfunction of neurological sub-systems or co-existing neurological conditions that are multi-factorial in etiology. As such, the initial assessment of the athlete with PCS should not be simply aimed at cataloging concussion symptoms, but rather combining elements of the clinical history and physical examination with results of other supplementary tests to elucidate the pathophysiological processes that govern persistent symptoms.

## Initial Assessment

A careful and complete clinical history is perhaps the most important component of the initial assessment of athletes with PCS and provides important clues that help guide the physical examination and aids in the identification of the pathophysiology underlying persistent concussion symptoms. Initial injury details that are important to elicit from the athlete include the mechanism of injury, the presence of loss of consciousness, post-traumatic amnesia or seizure; concussion symptoms experienced at the time of injury, and initial and subsequent medical management of the injury. Pre-existing medical conditions that may impact concussion recovery and symptom duration include a history of previous concussions, non-specific or migraine headaches, ADHD or learning disorders, psychiatric conditions such as depressive or anxiety disorders, previous cervical spine injuries, and pre-existing neuro-ophthalomological conditions such as strabismus or convergence insufficiency. A history of other disorders that can account for presenting or persisting symptoms and that can influence return-to-play decision-making such as a history of seizure disorders, structural brain abnormalities (i.e., Chiari Malformations, arachnoid cysts), hematological conditions (i.e., anemia or platelet disorders), and infectious diseases (i.e., mononucleosis) should also be assessed. Assessment of previous and present school and social functioning is also important to help gauge the effects of persistent concussion symptoms on cognitive and emotional functioning. A family history of psychiatric disorders and migraine headaches may also predispose patients to certain post-concussion symptoms or conditions.

In addition to assessing the nature and magnitude of the patient’s persistent concussion symptoms, it is also important to identify the circumstances, environments, or activities that elicit or exacerbate these symptoms. In this way, symptoms that are exacerbated by physical exertion point to a persistent global metabolic brain injury while those that are not made worse with exercise are more likely to result from isolated neurological sub-system dysfunction or other co-existing conditions. Symptoms commonly reported by athletes with PCS include headaches, dizziness, sensitivity to light and sound, difficulty focusing or concentrating, sleep disturbance, and fatigue. Because some athletes, in particular children, may not have the vocabulary to express subtle aspects of their symptoms, it is important that physicians ask specifically about fixed or transient neurological deficits such as monocular blurred vision or transient weakness or numbness that can point to focal injury to central and peripheral nervous system structures (63–65).

In order to identify objective impairments responsible for persistent concussion symptoms, all PCS patients should undergo a complete neurological examination ideally performed by an experienced sports medicine physician, neurologist, or neurosurgeon. Although standardized concussion assessment tools have become available (3, 66), use of these tools in isolation is insufficient to evaluate patients with PCS. The physical examination should include assessment of cranial nerve, motor, sensory, reflex, cerebellar, gait, and balance testing. In addition, a focused vestibulo-ocular examination that includes objective testing of convergence, smooth pursuits, saccades, and the vestibulo-ocular reflex should be performed (67, 68). All patients should undergo a cervical spine examination that includes range of motion, palpation, and provocative ligament and cervical dizziness testing. Patients that report monocular visual symptoms should undergo careful testing of pupillary function, visual acuity, visual fields, color vision, as well as fundoscopy to rule out optic nerve or retinal pathology (65). Those that report a history of intermittent vertigo should undergo the Dix-Hallpike maneuver to test for benign paroxysmal positional vertigo (BPPV). For a more detailed approach to physical examination in concussion and PCS patients readers are referred to the following review (69).

## Supplemental Testing

### Diagnostic Imaging

Over the past 10 years, several well-validated clinical decision rules have been developed to help guide the judicious use of diagnostic imaging in patients with acute traumatic head and cervical spine injuries that are evaluated in the emergency room setting (70–74). Unfortunately there are no evidence-based guidelines to direct the use of neuroimaging in PCS patients. Although current expert opinion suggests that clinical neuroimaging studies contribute little value to the clinical management of concussion patients (3), the presence of abnormal and traumatic structural neuroimaging findings detected in PCS patients can have an important impact on clinical decision-making in select cases. As such, MRI of the brain should be considered in patients with abnormal CT findings, worsening or persistent symptoms, focal neurological deficits, and seizures but is otherwise not indicated in most PCS patients (64, 75). Techniques such as susceptibility-weighted and gradient echo imaging have been shown to offer enhanced detection of micro-hemorrhages over CT and conventional MRI sequences and are important to include in this population (76). More recently, advanced neuroimaging techniques have been applied to SRC patients including DTI, task-based and resting state functional MRI, quantitative CBF sequences, and cerebrovascular reactivity mapping (75, 77). Despite broadening our understanding of concussion pathophysiology, none of these tools are used in the routine clinical management of PCS patients. In some athletes presenting with PCS, persistent symptoms can be attributable to previously unrecognized structural injury or instability of the cervical spine that should be investigated by static and dynamic cervical spine x-rays or MRI including STIR sequences (63, 78). Patients who are diagnosed with co-existing structural spine or spinal cord pathology should be referred to a neurosurgeon or orthopedic spine surgeon for further evaluation and management.

### Neuropsychological Testing

Post-concussion syndrome patients who report prominent cognitive or emotional symptoms and those who experience deterioration in school performance or functioning should be considered for referral to a clinical neuropsychologist. Despite the widespread use of computerized neurocognitive tools to evaluate patients with acute concussion, neuropsychological assessment of athletes with PCS is best accomplished by the use of multiple test batteries that examine domains such as concentration, memory, attention, processing speed, and executive function and are tailored to the patient’s persistent symptoms and the time course of injury (79). Clinical neuropsychologists are the only health-care professionals who are equipped with the clinical training, experience, and armamentarium of neuropsychological testing instruments that can provide comprehensive assessment of neurocognitive functioning in this patient population (80). Not only can the results of formal neuropsychological testing help to quantify the impact of TBI on subtle aspects of brain functioning but they are often instrumental in confirming complete neurocognitive recovery and helping guide return-to-learn and return-to-play decision-making.^2^ In patients with a history of multiple SRCs or who develop PCS and achieve symptomatic recovery, formal neurocognitive testing can reveal persistent alterations in cognitive functioning that may in some cases lead the multi-disciplinary team to recommend retirement from future contact or collision sports (64). Such assessments are also very helpful in developing strategies for patients experiencing semi-permanent cognitive issues at school or work.

### Graded Aerobic Treadmill Testing

**Figure 1:**
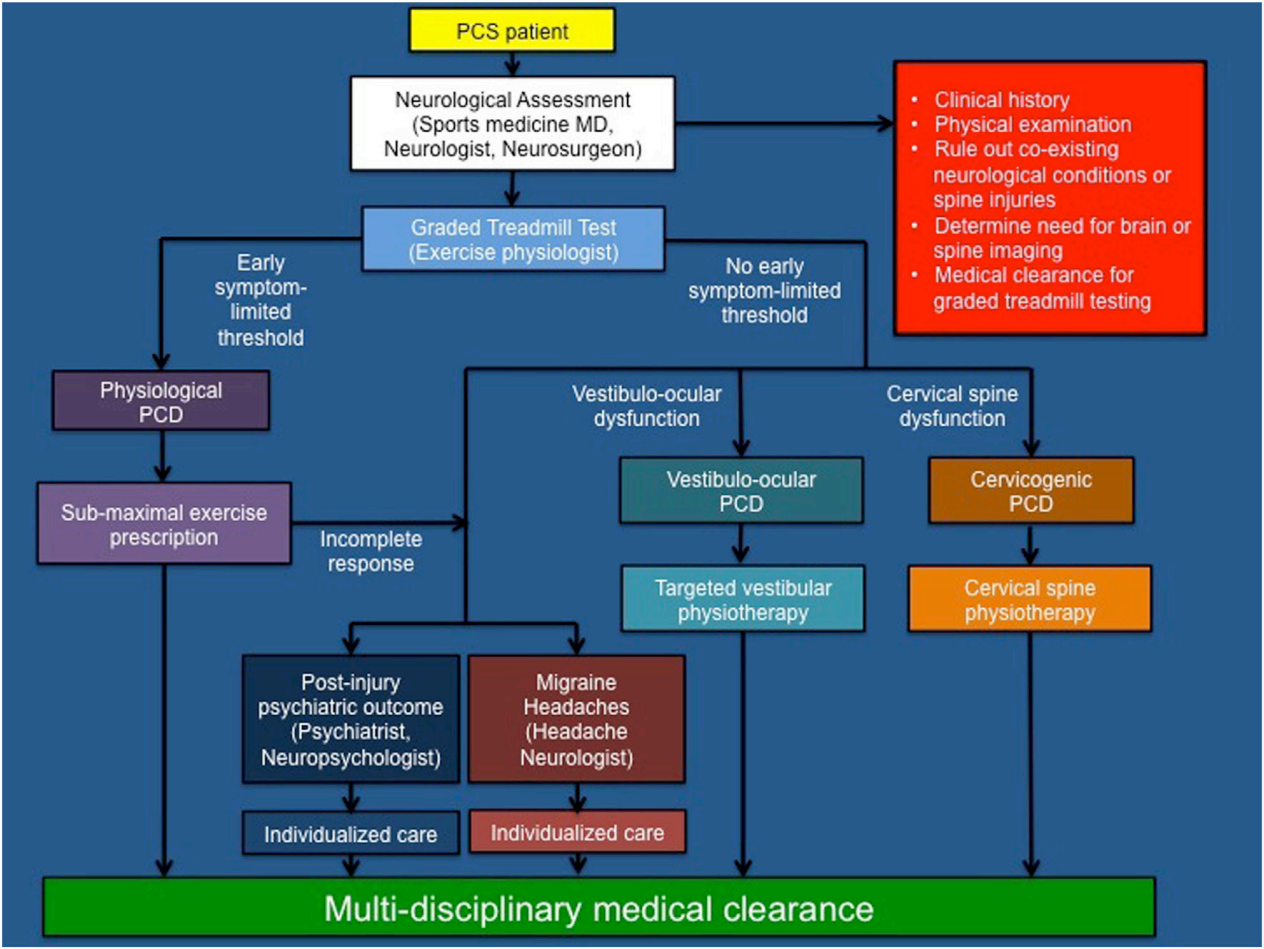
**Summary of multi-disciplinary pathophysiological approach to athletes with post-concussion syndrome**. PCS, post-concussion syndrome; PCD, post-concussion disorder; MD, medical doctor [modified with permission from original source (see footnote text 2), Journal of Neurosurgery Publishing Group].

Accumulating evidence suggests that graded aerobic treadmill testing is a safe, well-tolerated, and reliable tool to evaluate exercise tolerance and classify pediatric and adult SRC patients into operational PCS sub-types or disorders (7–9, 11, 80–82). This tool is valuable because previous work has demonstrated that evaluation of individual symptoms does not allow concussion patients to be appropriately classified into operational PCS sub-types or trajectories (82). Using a modified Balke protocol, PCS patients perform a standardized incremental aerobic treadmill test (the Buffalo Concussion Treadmill Test, BCTT). In this test, patients walk on a treadmill at a speed of 3.0–3.3 mph and 0% grade. The grade is increased by 1%/min during the first 15 min, after which the speed is increased by 0.2–0.4 mph/min. Patients are asked to rate their symptoms (using a Likert scale) and perceived exertion (using the Borg scale) every minute during testing while they undergo continuous heart rate monitoring. The test is terminated once a symptom-limited threshold or voluntary exhaustion is achieved (8). Because there are numerous medical contraindications to exercise testing that can exist in athletes, these tests are best undertaken by an experienced physical therapist, exercise physiologist or kinesiologist under the supervision of a physician (11). In general, patients who are asymptomatic or have concussion symptoms at rest and who exhibit an early symptom-limited threshold on treadmill testing are diagnosed with physiological post-concussion disorder (PCD), a condition initially proposed to be mediated by persistent alterations in global metabolic and cerebrovascular functioning. Patients who have persistent concussion symptoms that are not exacerbated by treadmill testing are diagnosed with either vestibulo-ocular PCD or cervicogenic PCD based on clinical evidence of dysfunction within the vestibulo-ocular and cervical spine neurological sub-systems. In our experience, physiological PCD patients typically reach an early symptom-limited threshold within 5–15 min of initiating treadmill testing. The threshold is defined by a ≥3 point change in symptom report on the Likert scale at any point during the test when compared with the patient’s pre-test symptom level (typically headache, head fullness or dizziness, where a point is given for an increase in a symptom and/or appearance of a new symptom). In general, those with vestibulo-ocular and cervicogenic PCD are able to exercise for 15–25 min without symptom exacerbation. In rare cases, patients with vestibulo-ocular and cervicogenic PCD can experience mild exacerbation of their symptoms that typically occurs towards the later stages of exercise when walking at higher inclines. In some cases, PCS patients may develop clinical features that meet the International Classification of Headache Disease 3beta (ICHD-3B) criteria for new or worsening migraine headaches (83), while others may develop post-injury psychiatric outcomes including mood disorders (84). Although some patients will demonstrate symptoms of multiple or overlapping PCDs, the following classification system provides a conceptual framework that allows physicians to identify the pathophysiological mechanisms that govern persistent concussion symptoms and informs the development of individually tailored rehabilitation programs (see Tables 1 and 2; Figure 1).

**Table 1:** **Summary of proposed pathophysiology, predominant symptoms, physical examination findings, graded treadmill testing results, treatment recommendations, important considerations and multi-disciplinary consultations for post-concussion disorders**.

	Physiological PCD	Vestibulo-ocular PCD	Cervicogenic PCD
Proposed pathophysiology	Persistent alterations in neuronal depolarization, cellular metabolism, and cerebrovascular physiology	Isolated dysfunction of central and peripheral components of the vestibulo-ocular neurological sub-system	Isolated mechanoceptive, nocioceptive, and proprioceptive dysfunction within the cervical spine neurological sub-system
Predominant symptoms	Mild to moderate, global, pounding headache at rest Dizziness, nausea, fatigue, drowsiness, light and sound sensitivity, irritability Symptoms elicited or exacerbated by reproducible levels of physical (and sometimes) cognitive activity	Mild to moderate headache and eye strain that is typically absent at rest but elicited or exacerbated by prolonged periods of reading, focusing, or time in complex visuospatial environments Intermittent blurred vision, diplopia, dizziness, fogginess, motion sensitivity, difficulty focusing or concentrating Intermittent vertigo during certain head positions	Mild to moderate, dull, occipital headache that is elicited or exacerbated by activities that require prolonged neck stabilization or movement Neck pain, stiffness, decreased range of motion, dizziness, fogginess, and postural imbalance
Physical examination findings	Normal physical examination Elevated resting heart rate Orthostatic changes in pulse and/or blood pressure accompanied by symptoms	Impaired convergence, accommodation, smooth pursuits, saccades, and vestibulo-ocular reflex Impaired balance and postural stability testing Positive Dix-Hallpike Maneuver (BPPV)	Decreased cervical lordosis and range of motion Sub-occipital and paraspinal neck tenderness Impaired cervical spine proprioception Positive cervical dizziness testing
Graded treadmill testing results	Early symptom-limited threshold	Patients typically do not experience an early symptom-limited threshold	Patients typically do not experience an early symptom-limited threshold
Treatment	Sub-maximal aerobic exercise prescription Targeted treatment of co-existing vestibulo-ocular dysfunction or cervical spine soft tissue injury	Targeted vestibular and vision therapy Otolith repositioning (BPPV) Sub-maximal aerobic exercise program to maintain aerobic fitness	Cervical spine manual therapy and proprioception re-training Gaze and postural stabilization exercises Sub-maximal aerobic exercise program to maintain aerobic fitness
Important considerations	Patients who do not achieve complete recovery with sub-maximal exercise prescription should be screened for migraine headaches and post-injury psychiatric outcomes	Must rule out co-existing neurological and neuro-ophthalmological conditions prior to graded aerobic treadmill testing and physiotherapy	Must rule out cervical spine structural injury or mechanical instability prior to graded aerobic treadmill testing and physiotherapy
Consulting multi-disciplinary specialists	Exercise physiologist or kinesiologist	Vestibular physiotherapist Neuro-ophthalmologist	Cervical spine physiotherapist

*PCD, post-concussion disorder; BPPV, benign paroxysmal positional vertigo [modified with permission from original source Ellis et al. (9), Taylor & Francis]*.

**Table 2:** **Summary of the clinical features, graded treadmill testing results, and treatment recommendations for the main types of headache that occur in post-concussion syndrome patients**.

	Physiological PCD	Vestibulo-ocular PCD	Cervicogenic PCD	Migraine
Clinical features	Mild to moderate, global, pounding headache at rest that is exacerbated by reproducible levels of physical (and sometimes) cognitive activity	Mild to moderate headache that is typically absent at rest but elicited or exacerbated by prolonged periods of reading, focusing, or time in complex visuospatial environments	Mild to moderate, dull, occipital headache that is elicited or exacerbated by activities that require prolonged neck stabilization or movement Moderate or severe, shock-like pain along the distribution of the occipital nerves that radiates to the top of the head or ears and is exacerbated by touch or head and neck movements (occipital neuralgia)	Paroxysmal attacks of unilateral, severe, throbbing or pulsating headaches associated with photo- and phonophobia, nausea, and occasional vomiting Provoked by stereotypical stimuli including bright lights, stress, dehydration, poor sleep, and certain foods. Headaches last 4–72 h after which patients typically experience headache-free periods
Graded treadmill testing results	Early symptom-limited threshold	Patients typically do not experience an early symptom-limited threshold	Patients typically do not experience an early symptom-limited threshold	Patients typically do not experience an early symptom-limited threshold
Treatment	Sub-maximal aerobic exercise prescription	Targeted vestibular and vision therapy	Cervical spine manual therapy and proprioception re-training Headache medications or occipital nerve injections (occipital neuralgia)	Sub-maximal aerobic exercise prescription Prophylactic and abortive headache medications

## Management

### Physiological Post-Concussion Disorder

Physiological PCD is a sub-type of PCS that is mediated by a persistence of the biochemical, autonomic, and cerebrovascular alterations that characterize acute concussion. Preliminary evidence to support this hypothesis includes the findings of alterations in resting CBF detected in adolescent and collegiate athletes that persist beyond symptomatic recovery (45, 47, 48) as well as regional alterations in resting CBF and cerebrovascular responsiveness observed in adolescents and adults with PCS (51–54). In addition, patients with PCS have been found to demonstrate higher heart rates at rest (49, 85, 86) and greater CBF during exercise suggestive of imbalances in autonomic nervous system activity (50).

Clinically, physiological PCD patients typically present with persistent concussion symptoms such as headaches, dizziness, fatigue, and sensitivity to light that are exacerbated by physical activity and point to a persistent mismatch between the metabolic needs of the injured brain and energy substrate delivery. In some cases, these patients are asymptomatic, and symptoms are only elicited by physical, and less often, cognitive activity. The physical examination of patients with physiological PCD is typically normal but an elevated resting heart rate may be observed. Diagnostically, patients with physiological PCD demonstrate evidence of exercise intolerance that is characterized by an early symptom-limited threshold on graded aerobic treadmill testing.

The mainstay of treatment for physiological PCD is tailored sub-maximal aerobic exercise prescription. Laboratory animal studies suggest that there is a therapeutic window during which time initiation of aerobic exercise can enhance neurological and neurocognitive recovery through the upregulation of brain-derived neuropeptides (87, 88). Clinical studies in both pediatric and adult concussion patients have shown a high rate of symptom improvement and complete recovery following individualized, tailored sub-maximal exercise prescription (see footnote text 2, 89, 90). This approach is also supported by one randomized controlled study in adolescent mild TBI patients with prolonged symptoms who demonstrated improvements in concussion symptoms following sub-maximal exercise prescription (91). Preliminary research suggests that neurological recovery in physiological PCD may be mediated by improvement or normalization of CO_2_ sensitivity, resting CBF, and cerebrovascular reactivity (50, 53, 54).

Accordingly, physiological PCD patients should be prescribed tailored sub-maximal aerobic exercise programs that target 80–90% of the heart rate achieved during graded aerobic treadmill testing, once a day for 20 min in duration, 5–6 days a week. Patients should undergo follow-up every 1–3 weeks for re-assessment at which time the physician may consider repeat treadmill testing and modification of the sub-maximal exercise prescription or transition into a sports-specific rehabilitation program. Importantly, physiological PCD patients may also demonstrate evidence of co-existing vestibulo-ocular dysfunction or cervical spine soft tissue injury that can be addressed concurrently with targeted rehabilitative strategies. Although, the vast majority of physiological PCD patients will achieve complete recovery with sub-maximal exercise prescription, patients who meet the ICHD-3B criteria for new or worsening migraine headaches or who develop post-injury psychiatric outcomes may experience an incomplete response. These patients should be managed on an individualized basis in collaboration with an experience headache neurologist or psychiatrist as discussed below.

### Vestibulo-Ocular Post-Concussion Disorder

Vestibulo-ocular PCD is a sub-type of PCS that is mediated by isolated dysfunction of the vestibular and oculomotor sub-systems. The oculomotor and vestibular systems are highly integrated systems composed of rich interconnections between cortical, sub-cortical, brainstem, spinal cord, cerebellar, and peripheral sensory organ structures that are responsible for maintaining balance, postural control, and gaze stability (67, 92). Accumulating evidence suggests that clinical features of vestibulo-ocular dysfunction at initial presentation following SRC are associated with an increased risk of prolonged recovery and the development of PCS (18, 19, 93). Clinical predictors of vestibulo-ocular dysfunction may include female sex, pre-injury depression, and post-traumatic amnesia, dizziness, blurred vision, and difficulty focusing at the time of injury (see footnote text 1). Patients with vestibulo-ocular dysfunction can present with heterogeneous and often overlapping deficits that manifest as objective impairments in convergence, accommodation, smooth pursuits, saccades, the VOR, or BPPV (67, 94). In some cases, objective evidence of central and peripheral vestibulo-ocular disorders may not be detectable, but patients are found to demonstrate symptom provocation on testing potentially pointing to more subtle vestibular pathology (66).

Clinically, vestibulo-ocular PCD patients typically present with dizziness, intermittent blurred vision or diplopia, difficulty focusing or concentrating, fogginess, motion sensitivity, postural imbalance, and headaches that are usually absent at rest but are elicited or exacerbated by prolonged periods of reading, focusing, or functioning within complex visuospatial environments. Patients with BPPV typically present with intermittent episodes of vertigo that are elicited by certain positions such as lying down or bending over. A comprehensive physical examination performed by a physician with training in TBI and neuro-ophthalmology (sports medicine physician, neurologist, neurosurgeon, and neuro-ophthalmologist) is especially important in this population. To evaluate the need for targeted rehabilitation in vestibulo-ocular PCD patients, physicians must rule out other structural or neurological conditions that can present with persistent visual disturbance (such as traumatic cranial nerve injury, retinal detachment, demyelinating disease etc.) and ensure that the patient’s subjective vestibulo-ocular complaints are attributable to objective findings on neuro-ophthalmological examination. Accordingly, the neuro-ophthalmological examination of PCS patients presenting with persistent visual disturbance should include testing of pupillary function, visual acuity, visual fields, resting ocular alignment, convergence, extra-ocular movements and smooth pursuits, saccades and the vestibulo-ocular reflex. The Dix-Hallpike maneuver should be completed in patients with vertigo and suspicion of BPPV. Although some abbreviated tools such as the vestibular oculomotor (VOMS) tool (66) may help identify patients with vestibulo-ocular dysfunction, there are indeed SRC patients that do not experience symptom provocation with these tests but do demonstrate objective findings that point to isolated vestibular pathology (i.e., VOR impairments). Patients with suspected cranial neuropathies or retinal pathology should undergo urgent referral to a neuro-ophthalmologist for more comprehensive evaluation. Previous neuroimaging studies should also be reviewed for intracranial findings (i.e., orbital or temporal bone abnormalities) that can contribute to visual or vestibular pathology. Diagnostically, patients with vestibulo-ocular PCD will present with subjective and objective evidence of vestibulo-ocular dysfunction but do not experience an early symptom-limited threshold on graded aerobic treadmill testing.

The treatment of vestibulo-ocular PCD must be individualized and aimed at rehabilitating the objective deficits and pathophysiological mechanisms underlying persistent symptoms. The principle goal of vestibular physiotherapy is to re-establish effective integration of the visual, vestibular, and somatosensory systems through a patient-tailored progressive vestibular exercise program (95). Specialized care of these patients should ideally be carried out by an experienced physiotherapist with advanced competency-based clinical training in post-traumatic central and peripheral vestibulopathies. To date, there is some empirical evidence to support improved outcomes in concussion patients treated with vestibular physiotherapy. For example, Alsalaheen (96) demonstrated improvement in vestibular symptom inventories and outcome measures in concussion patients treated with vestibular physiotherapy. Likewise, one randomized controlled trial in patients with SRC demonstrated enhanced recovery among those treated with cervicovestibular physiotherapy compared to a control group (97). In vestibulo-ocular PCD patients, whose symptoms are attributed to peripheral vestibular disorders, evidence can be taken from the general vestibular literature to direct treatment in these patients. As such, there is strong evidence to support the use of otolith repositioning techniques for patients with BPPV (98). There is also moderate to strong clinical evidence to support vestibular rehabilitation for patients with vestibular hypofunction (99, 100). Although there is low-quality evidence to support a clinical benefit of betahistine to treat vertigo and dizziness attributable to different vestibular pathologies (101), there are no prospective studies that have examined this pharmacological agent in concussion patients. In recent years, vision therapy has received increased attention as a potential therapeutic option to treat oculomotor disturbances, such as abnormalities of convergence, accommodation, smooth pursuits, and saccades. Some studies have reported improved outcomes in patients with mild TBI (102) and childhood convergence insufficiency (103) with vision therapy; however, there are no prospective studies that have examined the clinical benefit of vision therapy in concussion patients. Consequently, there remains an urgent need for future research to develop evidence-based indications for vestibular and vision therapy and evaluate the effect of these treatment options on objective findings and outcomes in patients with the different clinical manifestations of vestibulo-ocular PCD. Because vestibulo-ocular PCD patients do not experience an early symptom-limited threshold on graded aerobic treadmill testing, these patients should be advised to continue to exercise as tolerated to prevent aerobic deconditioning.

### Cervicogenic Post-Concussion Disorder

Cervicogenic PCD is a sub-type of PCS that is mediated by isolated dysfunction of the cervical spine neurological sub-system. The cervical spine is the most mobile segment of the spine and contains a complex network of muscle and joint mechano-, proprio-, and nocioceptive fibers that transmit important sensory information from the cervical spine to the spinal cord, brainstem, and cerebellum (104). These interconnections form specialized reflexes including the cervicocollic, vestibulocollic, and cervico-ocular reflexes that help mediate head, gaze, and neck position sense, and stabilization during rapid head movements. Biomechanical forces imparted to the brain at the time of SRC can also be transmitted to the cervical spine resulting in a whiplash-type injury and cervicogenic headaches that are mediated by a combination of local inflammation, central sensitization, and sensory and autonomic pathway dysfunction (105, 106). Trauma or persistent muscle spasm affecting the deep and superficial cervical and sub-occipital muscles can also lead to irritation and impingement of the sensory nerves that innervate the neck and posterior scalp leading to cervicogenic headaches and occasionally occipital neuralgia.

Clinically, cervicogenic PCD patients typically present with neck pain and stiffness, fatigue, and fogginess. Dizziness is also a common complaint among these patients and is usually absent at rest but elicited by activities that require prolonged neck stabilization or movement. The headaches described by cervicogenic PCD patients are often occipital in location, can radiate to the temples and eyes, and are frequently exacerbated by poor posture and neck-related activities such as weight training and running. In rare cases, patients can develop occipital neuralgia, which is characterized by severe episodes of shock-like pain that originate along the distribution of the greater and lesser occipital nerves, radiate to the top of the head and ears, and are exacerbated by touch or head and neck movements. Common physical examination findings include paraspinal and sub-occipital muscle tenderness and spasm, decreased cervical spine range of motion, and dizziness elicited during cervical dizziness testing. Tenderness and radiating pain can also often be elicited on palpation of the greater and lesser occipital nerves. In some cases, supplemental tools such as cervical joint position error can be useful to document cervical spine proprioceptive dysfunction. Patients that complain of co-existing neurological deficits such as upper or lower extremity weakness or numbness and those that demonstrate point tenderness on palpation of the central cervical spine should be considered for diagnostic imaging to rule out structural spinal pathology or instability prior to initiating any manual cervical spine rehabilitation (63, 64). Diagnostically, cervicogenic PCD patients do not experience an early symptom-limited threshold on graded aerobic treadmill testing.

The management of cervicogenic PCD is aimed at reducing local inflammation and muscle spasm, restoring range of motion, and re-calibrating communication between the cervical spine, vestibular, and oculomotor systems. These objectives are best accomplished by a tailored cervical spine rehabilitation program undertaken by an experienced physiotherapist that includes manual therapy, passive and active range of motion exercises, low velocity mobilizations, proprioceptive retraining, and exercises to strengthen the deep and superficial cervical musculature. Empirical support for this approach is provided by one randomized controlled trial demonstrating enhance recovery following cervicovestibular physiotherapy in SRC patients (97) and observational studies that have demonstrated improvements in pain and sensorimotor outcome measures among whiplash patients treated with multi-modal cervical spine rehabilitation (106–108). In addition, a recent review concluded that rehabilitation programs that employ cervical spine mobilization and manipulation techniques also provide benefit for cervicogenic headaches (109). Patients with severe whiplash-type injuries who develop persistent cervicogenic headaches and occipital neuralgia should be referred to an experienced headache neurologist for consideration of pharmacological management and occipital nerve injections (110). Because cervicogenic PCD patients do not experience an early symptom-limited threshold on graded aerobic treadmill testing, these patients should be advised to continue to exercise as tolerated to prevent aerobic deconditioning.

### Migraine Headaches

Although the vast majority of PCS patients will develop headaches that fit within the clinical framework of physiological, vestibulo-ocular, and cervicogenic PCDs, a small but important proportion will develop migraine headaches. Migraine headache is the most common primary headache disorder, affecting 15–20% of the general population with a peak incidence within the third and fourth decades of life (111). This condition has a strong hereditary basis with a positive family history frequently noted among patients (112). In the authors’ experience, the effect of SRC on pre-existing migraine headaches can be highly variable with some patients returning to their neurological baseline without exacerbation of their migraine headaches and others developing worsening migraines that require pharmacological intervention. The two most common scenarios that face physicians caring for athletes with SRC and migraine headaches are situations in which: (1) an athlete with no pre-injury history (but often a positive family history) of migraine or non-specific headaches sustains a SRC and develops new onset migraine headaches, and (2) an athlete with a pre-injury history of migraine headaches develops worsening migraine headaches after SRC. In some cases, the symptoms of PCS can be difficult to distinguish from migraine; however, certain features must be present to meet ICHD-3beta criteria for this condition (83). Because of these clinical subtleties, the management of both groups of athletes is ideally carried out in collaboration with an experienced headache neurologist. In general, migraine headaches are characterized by paroxysmal attacks of unilateral, severe, throbbing, or pulsating headaches that are frequently accompanied by photo- and phonophobia, nausea, and occasional vomiting. These headaches are typically provoked by stereotypical stimuli including bright lights, stress, dehydration, poor sleep, and certain foods. The headaches can last from 4 to 72 h after which patients typically experience headache-free periods. These headaches are distinguished from those experienced by physiological PCD patients that are typically global, pounding headaches that are mild to moderate intensity at rest, and along with other concussion symptoms are exacerbated by reproducible levels of physical exercise (Table 2).

Management of athletes with migraine headaches must be individualized. In our experience, some patients with new onset or worsening migraine headaches following SRC can be successfully treated with sub-maximal aerobic exercise prescription while some experience an incomplete response with this form of treatment (see footnote text 2). In these later cases, pharmacological management is often indicated and commonly includes the use of prophylactic medications such as tricyclic anti-depressants, beta-blockers, and anti-convulsants, symptomatic medications such as ibuprofen and acetaminophen and abortive medications such as triptans (113–115).

At present, there are no evidence-based guidelines to direct return-to-play decision-making in patients that have developed new onset migraine headaches or worsening migraines that have required pharmacological intervention. Because medications can mask headaches and other concussion-related symptoms, a multi-disciplinary approach to medical clearance including graded aerobic treadmill testing and formal neuropsychological testing is strongly advised in these patients.

### Post-Injury Psychiatric Outcomes

Another group of unique PCS patients that requires a multi-disciplinary approach is that presenting with primarily persistent emotional symptoms. Emotional symptoms such as sadness or depressed mood, nervousness or anxiety, irritability, fatigue, and difficulty sleeping are commonly reported among athletes following SRC and often resolve spontaneously along with other concussion symptoms with conservative management (116). However, in some cases, these symptoms can persist and lead to the development of post-injury psychiatric outcomes including novel post-injury psychiatric disorders, isolated suicidal ideation, or worsening symptoms of a pre-injury psychiatric disorder (84). Although the development of post-injury psychiatric outcomes following SRC is undoubtedly multi-factorial in etiology and impacted by individual pre-injury and post-injury factors, very few studies have examined these relationships in athletes with SRC (117–119). In one retrospective study of pediatric SRC patients presenting to a tertiary concussion program, post-injury psychiatric disorders were observed in 11% of patients and were more common among those that were female, had a pre-injury or family history of psychiatric illness, and who endorsed a greater burden of overall and emotional concussion-related symptoms at initial presentation (84). Given the complex nature of these cases, athletes with PCS and prominent mood-related symptoms should be managed by a multi-disciplinary team of healthcare professionals with clinical training and experience in TBI and psychiatry. A comprehensive clinical interview that provides an assessment of pre- and post-injury cognitive and mood-related functioning and incorporates validated age-appropriate clinical measures that screen for mood and psychiatric disorders is recommended for this population (120). Physicians should also screen for the use of alcohol and drugs that can precipitate, exacerbate, or prolong emotional symptoms of concussion. Overall, the pathophysiology of post-traumatic psychiatric outcomes remains poorly understood but may be mediated by a combination of pre-existing psychosocial factors as well as alterations in cerebral metabolism, neurotransmitter availability, and CBF (121–124).

The management of these athletes must be individualized and should take into consideration the impact of affective symptoms on social, school, and sports activities. Some patients may experience a symptom-limited threshold on graded aerobic exercise testing and benefit from sub-maximal exercise prescription and re-introduction of sports-specific activities. However, some patients may not achieve a symptom-limited response on graded aerobic treadmill testing or may experience an incomplete response to sub-maximal exercise. In these cases, a multi-modal approach employing pharmacological and behavioral interventions as well as close follow-up with a psychiatrist is recommended. Importantly, because of the extensive overlap between the clinical manifestations of PCS and pre- and post-injury mood disorders, we recommend a multi-disciplinary approach to medical clearance and return-to-play decision-making in this patient population (64).

## Future Directions

As illustrated in this report, significant advances in basic science, neuroimaging, exercise science, and clinical research have broadened our understanding of the heterogeneous pathophysiological processes responsible for the clinical manifestations of PCS. Despite this progress, a number of persistent key questions and knowledge gaps remain to be addressed. In the future, clinicians caring for athletes with PCS would benefit from research aimed at addressing the following questions:

- What is the natural history of autonomic nervous system, cardiovascular, and CBF alterations in patients with acute SRC and physiological PCD?
- What tools can be used in the clinical setting to measure autonomic nervous system, cardiovascular, and CBF dysfunction in PCS patients and help confirm physiological recovery of these parameters?
- What is the ideal timeframe to undertake graded aerobic treadmill testing and initiate sub-maximal aerobic exercise treatment following acute SRC?
- What is the natural history of vestibulo-ocular dysfunction following acute SRC and what are the evidence-based clinical indications for vestibular and vision therapy?
- What are the ideal clinical tools to assess central and peripheral pathophysiological mechanisms responsible for vestibulo-ocular dysfunction in acute SRC and PCS patients?
- Does targeted vestibular and vision therapy lead to enhanced recovery of the subjective and objective manifestations of persistent vestibulo-ocular symptoms compared to conservative management?
- What is the natural history or behavior of migraine headaches following SRC?
- What is the effect of sub-maximal exercise treatment and pharmacological intervention on the pathophysiological mechanisms governing post-traumatic migraine headaches?
- What are the biological, psychological, and social contributors to mental health-related outcomes following SRC?
- How does patient age impact the timing of multi-disciplinary active rehabilitation following acute SRC and PCS?
- What is the ideal multi-disciplinary approach to confirming neurocognitive and physiological recovery in athletes with PCS?
- What are the qualified roles and responsibilities of specific healthcare providers in the management of athletes who present with prolonged clinical manifestations of SRC and TBI?
- What are the clinical indications to advise athletes to retire from future contact and collision sports in the setting of multiple concussions or prolonged recovery?

## Conclusion

Athletes who present with persistent concussion symptoms and PCS represent a challenging patient population whose care must be individualized and is best carried out by a multi-disciplinary team of experts with clinical training and experience in TBI. Using the evolving clinical approach presented here, features of the clinical history and physical examination, and results from graded aerobic treadmill testing can be used to identify the unique and often overlapping pathophysiological mechanisms governing persistent symptoms and help formulate targeted rehabilitation programs that promote clinical recovery and return to sports. Future prospective studies employing multi-modal laboratory and neuroimaging techniques are needed to develop reliable biomarkers that can help identify the temporal profile of these pathophysiological mechanisms and confirm their recovery in the clinical setting. Work is also needed to identify the optimal period to initiate targeted rehabilitative therapies across age groups and establish empirical evidence to support these interventions.

## Author Contributions

Conception and design of the work (ME, JL, and BW). Drafting the work and revising it critically for important intellectual content (ME, JL, and BW). Final approval of the version to be published (ME, JL, and BW). Agreement to be accountable for all aspects of the work ensuring that questions related to the accuracy or integrity of any part of the work are appropriately investigated and resolved (ME, JL, and BW).

## Conflict of Interest Statement

The authors or their affiliated institutions have not received any payment or services from a third party for any aspect of the submitted work. The authors have no other disclosures or conflicts of interest.

## Footnotes

^1^Ellis M, Cordingley D, Vis S, Reimer K, Leiter J, Russell K. Clinical predictors of vestibulo-ocular dysfunction in pediatric sports-related concussion. *J Neurosurg Pediatr* (Forthcoming).

^2^Cordingley D, Girardin R, Reimer K, Ritchie L, Leiter J, Russell K, et al. Graded aerobic treadmill testing in pediatric sports-related concussion: safety, clinical use, and patient outcomes. *J Neurosurg Pediatr* (Forthcoming).
